# Five-Year Survival Rate of Bladder Cancer in Iran during 2001-2007

**DOI:** 10.30699/ijp.2020.118375.2287

**Published:** 2020-10-10

**Authors:** Maryam Khayamzadeh, Fereshte Aliakbari, Zahra Zolghadr, Majid Emadeddin, Mahsa Ahadi, MohammadEsmaeil Akbari, Amir Reza Abedi, Shahrzad Nematollahi, Jalil Hosseini

**Affiliations:** 1 *Cancer Research Centre, Shahid Beheshti University of Medical Sciences, Tehran, Iran*; 2 *Men’s Health and Reproductive Health Research Center, Shahid Beheshti University of Medical Sciences, Tehran, Iran*; 3 *Department of Biostatistics, School of Allied Medical Sciences, Shahid Beheshti University of Medical Sciences, Tehran, Iran*; 4 *Urology Department, Shohada-ye Tajrish Hospital, Shahid Beheshti University of Medical Sciences, Tehran, Iran*

**Keywords:** Urinary bladder neoplasm, Survival analysis, Iran, Disease registry

## Abstract

**Background & Objective:**

Bladder cancer is the fourth most common cancer in men and the most common cancer in women, comprising 8% of all males and 3% of female tumors. The present study aimed to estimate the five-year survival rates of bladder cancer in Iran.

**Methods:**

Information on 3,337 registered cases of bladder cancer was obtained from the Office of National Cancer Registry in the Ministry of Health and Medical Education (MOH & ME). A telephone survey was conducted to gather additional information, such as survival status, demographic, and clinical profile. Kaplan–Meier estimates of five-year survival rates were calculated according to the age of diagnosis, gender, pathological type, and provincial pole.

**Results and Conclusion:**

Overall five-year survival rate was 77%. According to the pathologic type, five-year survival rates were 81%, 66%, 81%, 42%, 77%, and 82% in low-grade urothelial carcinoma, high-grade urothelial carcinoma, adenocarcinoma, undifferentiated carcinomas, Squamous Cell Carcinomas (SCCs), and other tumors, respectively. Additionally, those tumors were 93%, 88%, 81%, 64%, and 44% among patients whose average ages at diagnosis were < 50, 50–59, 60–59, 70–79, and > 80 years old, respectively. Our study revealed that age and histological type were the major prognostic factors for survival in patients with bladder cancer. Therefore, given the histologic features of the tumor and patients with advanced age, a continuous screening would be highly warranted.

## Introduction

Bladder cancer accounts for 3% of all cancer morbidities (1, 2). Men typically have a three-times higher risk of bladder cancer contraction and diagnosis (3, 4). The most recent national cancer report indicated that the age-specific incidence rate (ASIR) of bladder cancer is nearly four-fold higher among Iranian men (13.03 vs. 3.32 in females per 100,000 population) (5, 6). Approximately two-thirds of new cases of bladder cancer are diagnosed with superficial histology, making it the most common type of bladder cancer (7, 8). 

Despite being fatal, survival rates for invasive bladder cancer have been largely unchanged since the 1990s, mainly due to delays in the treatment of muscle-invasive bladder cancer (9-11). The American Cancer Society has estimated that 5-, 10-, and 15-year survival rates for patients with bladder cancer are 77%, 70%, and 65%, respectively. However, the survival of bladder cancer patients depends heavily on many factors, including diagnosed type and the stage of bladder cancer, age, gender, high-risk behaviors (such as tobacco smoking and illicit drug use), and occupational hazards (12-15).

The Islamic Republic of Iran has experienced several cancer registry initiatives during the last decade (16, 17). Despite the well-performed cancer registry system, little epidemiological evidence exists on bladder cancer morbidity and mortality in Iran, whereas prevention and control of bladder cancer require valid information from population-based or pathology-based cancer registries (18, 19). Therefore, this study was conducted to estimate the survival rate of bladder cancer in Iran during 2001–2007.

##  Materials and Methods

Data of patients with primary bladder cancer (*n* = 16,702) were collected from the Office of National Cancer Registry in the Ministry of Health and Medical Education (MOH&ME) for the period of 2001–2007. An additional telephone survey by trained interviewers was conducted to collect data, including survival status, demographic characteristics, age at diagnosis, pathological findings, and clinical profile. According to the study protocol, three telephone calls within two consecutive weeks were considered sufficient to collect information. 

For statistical analyses, age at diagnosis was categorized into five age groups: less than 50, 51–60, 61–70, 71–80, and +80 years. Pathological type of bladder cancer was dichotomized, and provincial pole was categorized into nine geographical regions based on the similar socioeconomic status of regions:* Mazandaran* (Universities of Mazandaran, Gilan, Golestan, Semnan, and Babol), *Shiraz* (Universities of Shiraz, Bushehr, Bandar Abbas, Kohgeloyeh, Jahrom, and Fesa), *Kermanshah* (Universities of Kermanshah, Kordestan, Hamadan, and Ilam), *Ahwaz* (Universities of Ahwaz and Lorestan), *Tabriz* (Universities of Tabriz, West-Azarbayejan, Ardabil, and Zanjan), *Tehran* (Universities of Tehran, Markazi, Gahzvin, and Qom), *Kerman* (Universities of Kerman, Zahedan, Zabol, and Rafsanjan), *Khorasan* (Universities of Khorasan-Razavi, South Khorasan, North Khorasan, Sabzevar, Shahroud, and Gonabad), and *Isfahan* (Universities of Isfahan, Yazd, Chaharmahal, and Kashan). 

Using survival analysis set up, Kaplan–Meier survival rates were calculated according to age at diagnosis, gender, pathological type, and provincial pole. All the analyses were done using STATA 14 (StataCorp. 2015. Stata Statistical Software: Release 14. College Station, TX: StataCorp LP), and probability values (P-values) less than 0.05 were considered statistically significant.

## Results

From 2001 to 2007, registration information of 7,686 cases was retrieved from the national cancer registry system. Of those, successful telephone contact was performed for 3,946 cases (51%). Finally, information of 3,337 patients (response rate = 43%) was collected. Further, 83.4% of the patients (*n* = 2783) were males, and the rest (*n* = 554, 16.6%) were females (see Table 1). The highest proportion of the cases were diagnosed with bladder cancer at the ages of 70–79 years (28.2%), while low-grade urothelial carcinoma (*n* = 2331, 69.9%) was the predominant histological type, followed by high-grade urothelial carcinoma (*n* = 879, 26.4%; see Table 1). The distribution of the pathology types according to age and gender is presented in Table 2. 

**Table 1:** General characteristics of bladder cancer patients registered in the cancer registry in Iran during 2001–2007

Variable	Category	Frequency	Relative frequency
Gender	Male	2783	83.34
	Female	554	16.5
Age at diagnosis (years)	≤50	501	15
	50-59	644	19.3
	60-69	798	23.9
	70-79	942	28.2
	>80	452	13.5
Pathologic type	Low Grade	2331	69.9
	High Grade	879	26.4
	Papillary	33	1
	Undifferentiated	18	0.5
	SCC	48	1.4
	Others	28	0.8
Provincial pole	Mazandaran	372	11.1
	Shiraz	380	11.4
	Kermanshah	312	9.3
	Ahvaz	226	6.8
	Tabriz	253	7.6
	Tehran	617	18.5
	Kerman	166	5
	Khorasan	294	8.8
	Esfahan	715	21.4

**Table 2:** Distribution of registered pathology types of bladder cancer based on age and gender, 2001–2007

Variable	Category	Pathology type						Total
		TCC-High Grade	TCC-Low Grade	SCC	Adenocarcinoma	Others	undifferentiated	
Age group	<50	1856 (67.6%)	788 (28.7%)	41 (1.5%)	23 (0.8%)	21 (0.8%)	17 (0.6%)	2746 (100%)
	>50	91 (15.4%)	475 (80.4%)	7 (1.2%)	10 (1.7%)	7 (1.2%)	1 (0.2%)	591 (100%)
Gender	Male	1922 (69.1%)	763 (27.4%)	41 (1.5%)	20(0.7%)	22 (0.8%)	14 (0.5%)	2782 (100%)
	Female	408 (73.6%)	116 (20.9%)	7 (1.3%)	13 (2.3%)	6 (1.1%)	4 (0.7%)	554 (100%)

**Fig. 1:**
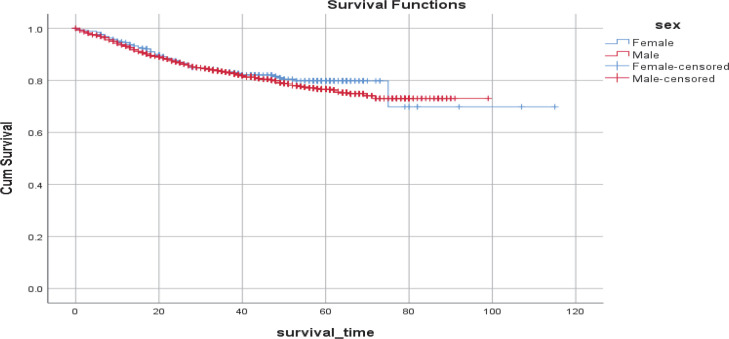
Kaplan–Mayer estimates of the overall survival of bladder cancer by gender

**Fig. 2:**
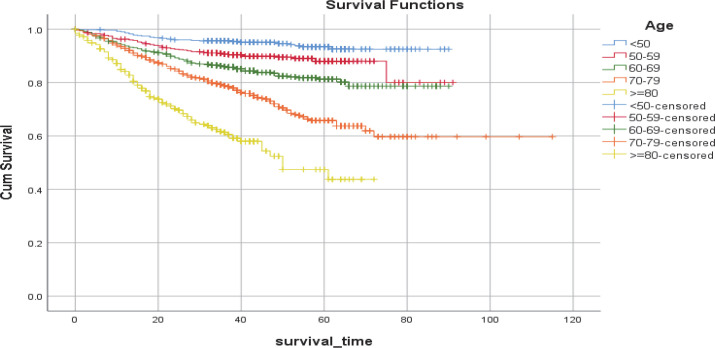
Kaplan–Mayer estimates of the overall survival of bladder cancer by age

The overall five-year survival rate of bladder cancer was estimated as 77% (Figure 1). Mean (SD) survival time (in months) was 92.45 (1.00); however, it was 92.30 (4.08) in women and 80.55 (0.79) in men. The difference in survival time between men and women was not statistically significant (*P*=0.47). Overall mean survival time and five-year survival decreased significantly as patients were diagnosed at older ages. Accordingly, mean (SD) survival time was 85.58 (0.80), 81.58 (1.53), 76.9 (1.11), 82.74 (2.12), and 46.07 (1.66) months, and five-year survival were 93%, 88%, 81%, 64%, and 44% among patients whose average ages at diagnosis were < 50, 50–59, 60–69, 70–79, > 80 years old, respectively (the P-value for the log-rank test was < 0.001; see Figure 2). 

The gender-specific pattern of mean survival time and five-year survival with respect to age also yielded statistically significant results. Accordingly, the male-to-female ratio of mean survival times in age at diagnosis of less than 50, 50–59, 60–69, 70–79, and +80 years old were 1.34, 1.18, 1.01, 0.82, and 1.02, respectively (all the P-values were < 0.001; Figure 2). 

**Fig. 3:**
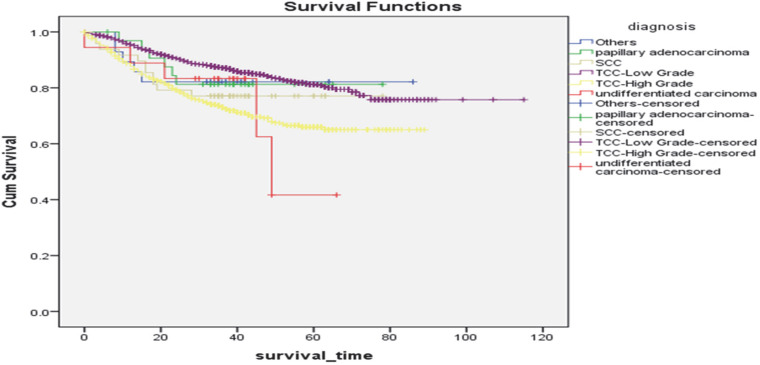
Kaplan–Mayer estimates of the overall survival of bladder cancer by pathological type

Overall mean (SD) survival time and five-year survival according to pathology types were 95.9 (1.2), 65.8 (1.2), 66.7 (4.1), 48.9 (5.7), 63.1 (3.9), and 72.5 (5.4) months, and 81%, 66%, 81%, 42%, 77%, and 82% in low-grade urothelial carcinoma, high-grade urothelial carcinoma, adenocarcinoma, undifferentiated carcinomas, Squamous Cell Carcinoma (SCCs), and other tumors, respectively, (*P*<0.001; Figure 3). More specifically, the male-to-female ratio of survival times for low-grade urothelial carcinoma, high-grade urothelial carcinoma, adenocarcinoma, undifferentiated carcinoma, SCCs, and other tumors were 0.88, 1.09, 1.06, 1.21, 1.70, 1.29, respectively. 

Among male patients, five-year survival rates were estimated as 81%, 67%, 75%, 29%, 80%, and 81% in low-grade urothelial carcinoma, high-grade urothelial carcinoma, adenocarcinoma, undifferentiated carcinoma, SCCs, and other tumors, respectively. Among female patients, the five-year survival rates were estimated as 84%, 62%, 92%, 75%, 57%, and 83% for low-grade urothelial carcinoma, high-grade urothelial carcinoma, adenocarcinoma, undiff-erentiated carcinoma, SCCs, and other tumors, respectively (*P*<0.001 for mean survival time). 

With respect to the provincial poles, the highest survival time (in months) was reported from the Isfahan pole (92.1±1.8), and the lowest survival time was reported from the Khorasan pole (54.3±1.2). According to the provincial poles, the difference among mean survival times did not yield statistically significant results (*P*=0.922).

## Discussion

Globally, more than 60% of all bladder cancer cases and half of all the 165,000 bladder cancer deaths occur in less developed countries (20, 21). Globally, there is a higher proportion of male cases so that three-quarters of all bladder cancer cases is reported to occur in men. Bladder cancer is the sixth common malignancy in Iran with an estimated ASIR of 8.4 per 100,000 population and considered as the second prevalent cancer among Iranian men (20, 22).

This study demonstrated that the risk of bladder cancer death varies strongly by age, sex, and geographic location. The lowest survival (equivalent to the highest risk of death) was observed in patients older than 80 years old in both genders and across all the provinces. This finding is consistent with other studies of which the incidence rates of bladder cancer increases by age, specifically in men (1, 23). Our study revealed that the risk of bladder cancer death was higher in men than in women, which is consistent with other studies in the world. 

Disparities in the incidence and mortality of bladder cancer according to gender can be attributed to lifestyle factors such as tobacco and opium use (24, 25). The results of the STEP wise approach to surveillance (STEPs) in Iran showed that the prevalence of current tobacco use in Iran has increased from 12% in 2000 to nearly 18% in 2009, while men were more likely to be current cigarette smokers than women (26, 27). Occupational exposures to hazardous substances (such as aromatic amines and other painting chemicals, rubber, or aluminum industries) are other well-known risk factors for the etiology of bladder cancer f (14, 15, 28). These exposures may greatly explain the observed gender differences in our study. Although women have made great strides in the workplace, developing countries are striking with inequality at workplaces. Such inequality is hardly unique to Iran. 

We found that five-year survival for bladder cancer consistently increased by advanced age. This finding is generally in accordance with other researches indicating higher survival in younger men and women and decreased trend of survival with increasing age (29). Based on our results, increased life expectancy in Iran and inherently poor prognosis of bladder cancer in elderlies might explain the observed pattern of mortality in advanced years. In our study, patients with high-grade bladder cancer had higher mortality rates than patients with low-grade bladder cancer. Mortality rate was higher in patients whose bladder cancer invaded the detrusor muscle, which is consistent with other studies (30). 

Karim Chamie *et al.* reported that 33% of patients with recurrent high-grade non-muscle-invasive bladder cancer will progress to having muscle-invasive bladder cancer, 40% of whom will die of bladder cancer (31). Different access to healthcare services can explain the difference in bladder cancer incidence and survival across countries (12). Better survival in countries with the higher Human Development Index (HDI) is most likely due to greater access to healthcare and hence better therapeutic options (16). In terms of the survival rate of bladder cancer, our study showed that the Khuzestan pole had the lowest and the Isfahan pole had the highest survival. Iran falls among countries with high HDI based on the 2009 Human Development Report. 

However, the distribution of HDI components varies greatly across the provinces in Iran, and the difference between high- and low-developed provinces increased in 2009. In the same year, Tehran province was ranked first (HDI = 0.791), while Sistan and Baluchistan province was at the bottom of the list (HDI = 0.601) (32). Therefore, the observed discrepancy among provinces in terms of survival might be partially explained by the various levels of HDI within the provinces. Overall, it seems that HDI cannot entirely predict the mortality of bladder cancer in Iran, and other important factors should be considered.

Our study used registration data from the population-based cancer registry system, which guarantees the wide generalizability of the included patients. However, the necessity for up-to-date information on the survival of bladder cancer might be jeopardized by considerable missing information on clinical data of registered patients. Moreover, due to incomplete information, we could not estimate the incidence of bladder cancer; therefore, estimated survival rates in our study should be interpreted with caution.

## Conclusion

Our study revealed that age and histological type were the major prognostic factors for survival in patients with bladder cancer. Therefore, given the histologic features of the tumor and patients with advanced age, a continuous screening would be highly warranted. 
